# Differential impact of various in-class physical exercise interventions on cognitive function and mathematics achievement in primary school children

**DOI:** 10.1038/s41598-026-45347-x

**Published:** 2026-03-24

**Authors:** Christian Leukel, Benedikt Lauber, Juliane Leuders, Sven Hertel, Wolfgang Taube

**Affiliations:** 1 University of Education Freiburg, Kunzenweg 21, 79117 Freiburg, Germany; 2 Bernstein Center Freiburg, University of Freiburg, Freiburg, Germany; 3 Department of Neurosciences and Movement Science, Faculty of Science and Medicine, University of Fribourg, Fribourg, Switzerland; 4 State Teacher Training Center, Lörrach, Germany

**Keywords:** Cognitive function, Balance training, Brain break, Selective attention, Human behaviour, Health care

## Abstract

Physical activity is increasingly recognized for its dual role in enhancing both physical health and cognitive development. This study compared the effects of three types of in-class interventions—CARDIO (aerobic/anaerobic exercises), BALANCE (balance training), and MENTAL (cognitive training without physical activity)—on cognitive function and mathematics achievement among primary school students, including a subsample with lower concentration scores. A total of 157 students from the 2nd and 3rd grades were assigned to one of the intervention groups. The interventions were conducted daily for 15 min over a 5-week period. Cognitive function was assessed using the KoKi concentration test, and academic achievement in mathematics was evaluated with a curriculum-based assessment. Concentration levels were increased in all groups, but only the BALANCE and MENTAL groups showed significant improvements in both easy and complex math exercises. In students with lower concentration scores, greater gains in simpler math tasks were observed for BALANCE and MENTAL groups. Both physical (BALANCE) and non-physical (MENTAL) breaks were associated with improvements in concentration and mathematics performance. Considering widespread sedentary behavior, physically active breaks may provide added health-related benefits.

## Introduction

Physical activity is recognized as a critical component of comprehensive school-based education, playing a pivotal role in both physical health and cognitive development^1^. Research indicates that regular participation in physical activities can result in improved cardiovascular health, increased muscular strength and better weight management among children^2^. Moreover, evidence shows that physical activity can significantly enhance cognitive function and academic achievement^3^. Cognitive function refers to basic mental processes, such as executive functions, which can impact academic achievement^4^. Academic achievement is defined as a child’s performance in school-related learning tasks, often assessed through standardized tests, progress monitoring tools, and self-reported perceived academic competence^4,5^. Consequently, integrating structured physical activity programs into the school curriculum is not only essential for fostering lifelong health habits^4^ but also for increasing the academic outcome of children^6,7^.

Recent studies have emphasized the significance of incorporating physical activity into the classroom as a targeted strategy within the school curriculum to provide a supportive learning environment^8^. Given that children dedicate a significant portion of their day to sedentary classroom tasks, researchers advocate for exploring interventions that not only counteract the negative impacts of prolonged sitting but also boost cognitive function and academic achievement through physical activity. According to Watson et al.^8^, classroom-based physical activity interventions can be divided into three main categories: Active breaks, which involve brief bursts of physical activity in academic lessons; curriculum-focused active breaks, incorporating physical activities related to the curriculum; and physically active lessons, where movement is integrated into non-physical education subjects like math to promote both, learning and movement simultaneously. Various studies have highlighted positive effects of active breaks on cognitive function and academic achievement^9,10^. Active breaks have been shown to have positive effects on concentration and selective attention, while results regarding sustained attention and information processing varied across studies^11–17^. Incorporating active breaks into school lessons has also been linked to improvements in academic achievement. However, the results across studies are inconsistent and appear to depend on the type of assessment used. While standardized tests generally did not show significant effects, tests aligned with the curriculum and reflecting the student’s current learning progress demonstrated positive outcomes^12,14,18–21^.

Despite the wealth of research on the effects of active breaks on cognitive function and academic achievement, there is a notable gap in the literature regarding comparisons between different forms of classroom-based physical exercise programs. While aerobic/anaerobic exercise training has been extensively studied and demonstrated to improve both cognitive activity and academic achievement^8–10^, alternative forms of exercise such as balance training may offer equally or even greater improvements in cognitive function and academic performance. Balance training challenges an individual’s ability to maintain equilibrium, through exercises such as standing on wobbling boards and soft mats^22^. Research in older adults has shown that balance training can significantly improve working memory and selective attention^23^. Studies indicate that balance training can upregulate inhibitory mechanisms reliant on gamma-aminobutyric acid (GABA) in both young and older adults^24,25^. These GABAergic processes play a crucial role in attention and hyperactivity, and mutations in genes associated with GABA function have been observed in individuals with attention deficit hyperactivity disorder (ADHD)^26^. Therefore, balance training is a promising form of classroom-based physical activity that may provide unique cognitive benefits. Since balance training enhances GABA-dependent inhibitory mechanisms it is reasonable to assume that children with lower selective attention abilities could particularly benefit in terms of cognitive function and academic achievement.

Furthermore, purely cognitive (i.e. non-physical) forms of interventions such as mindfulness-based programs have been proposed to improve cognitive function, attention and academic achievement in both healthy children^27^ and children with ADHD^28^. However, the effect sizes are small to moderate and the overall study quality in these meta-analyses is considered poor, with some studies also incorporating physical yoga exercises into the intervention, questioning the purely cognitive origin of the measured effects^28^. In line with this, a study directly comparing the influence of balance training with relaxation training on cognitive functions reported significantly improved memory and spational cognition exclusively after balance training with no effects in the relaxation group^29^.

The goal of the present study was to compare the effects of three different types of active break in-classroom interventions on cognitive function and academic achievement with a specific focus on arithmetic learning (i.e., subtraction (2nd grade) and multiplication (3rd grade) with one- and two-digit numbers) in mathematics education among primary school students. This is a fitting indicator for improvements in academic achievement because solving subtraction and multiplication tasks rely on fundamental cognitive processes such as selective attention, working memory and inhibitory control (e.g., keeping intermediate results in mind, and flexibly switching between calculation strategies)^30^. The task structure further shapes cognitive demands: simple single-digit problems can often be solved through automated retrieval, whereas two-digit subtraction and multiplication require the coordination of multi-step procedures (e.g., regrouping, carrying, handling zeros) and thus place higher demands on cognitive functions^30^.

The three different types of interventions in the present study were carried out over a 5-week period, consistent with previous studies on the effects of active break interventions^8^. The majority (90%) of these active breaks took place at the beginning of mathematics lessons. Two of these interventions involved physical activities: (i) moderate aerobic and anaerobic exercises (CARDIO), and (ii) balance training (BALANCE). The third intervention focused solely on cognitive training (“MENTAL”) and did not include physical activity. The MENTAL intervention consisted of structured desk-based exercises targeting selective attention and working memory (e.g., numerical and lexical recognition, listening and recall tasks), combined with brief relaxation and mindfulness elements. The choice of these training regimes (CARDIO, BALANCE, MENTAL) was guided not only by their theoretical relevance but also by their high feasibility and practicality for everyday classroom implementation, ensuring that teachers could easily integrate them into the classroom routine. The effects of these interventions on cognitive function were evaluated using standardized test instruments (KoKi^31^). Academic achievement was assessed through curriculum-based assessment specifically designed for the student cohort in our study.

Building on the findings of previous studies regarding the impact of balance training on GABAergic inhibitory mechanisms^24,25^, as well as the established link between selective attention deficits (such as those seen in ADHD) and altered GABA regulation, we further investigated whether children with lower selective attention would particularly benefit from balance training in terms of cognitive function and academic achievement.

## Materials and methods

### Subjects

**Fig. 1:**
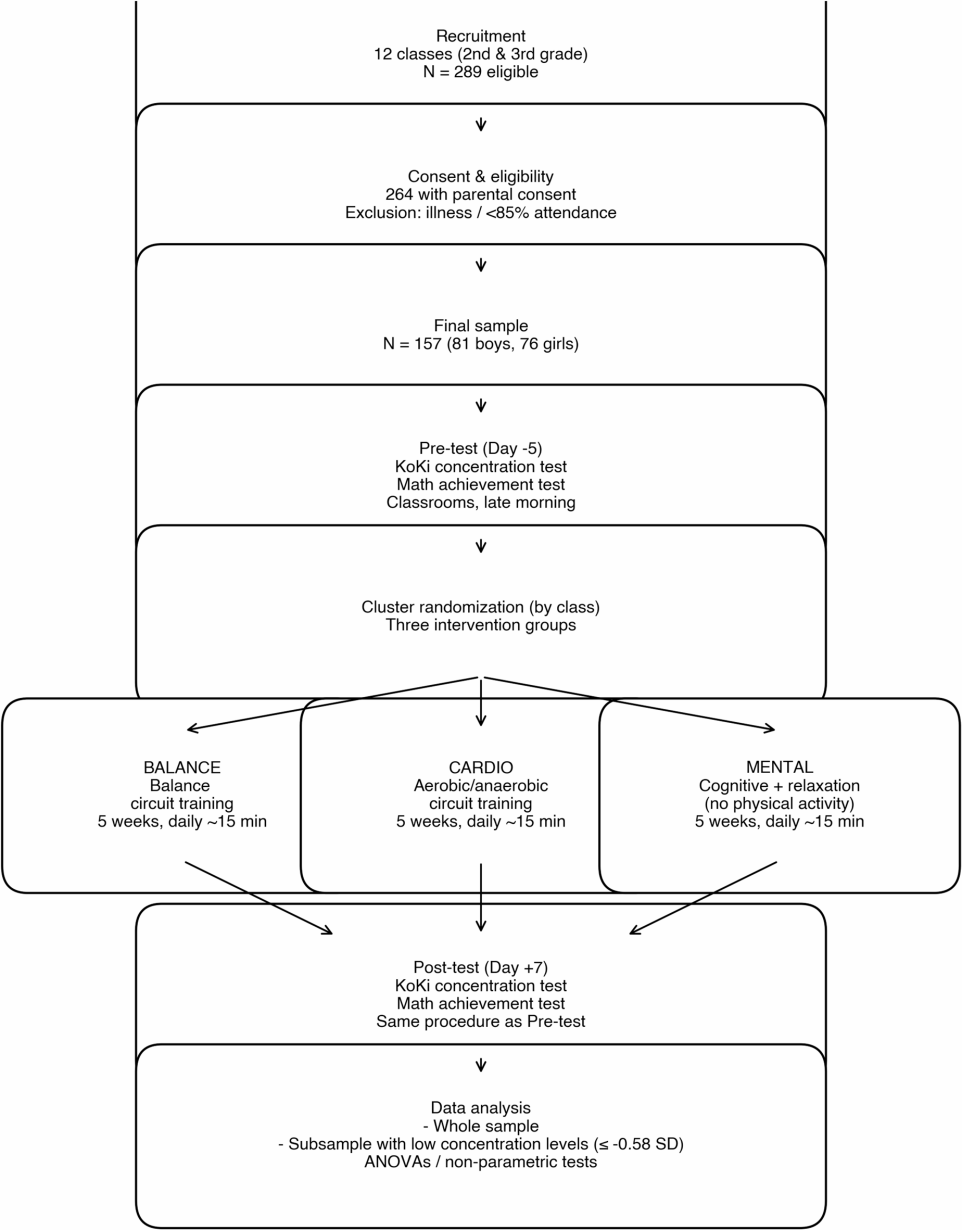
Summarizes all stages of the study, beginning with the recruitment of 12 classes (2nd and 3rd grade), followed by consent procedures, eligibility screening, and the final sample included in the analyses. After the pre-test assessment of cognitive function and academic achievement, classes were cluster-randomized into one of three active intervention groups (BALANCE, CARDIO, MENTAL). All interventions were conducted daily for 5 weeks, with sessions lasting approximately 15 min. After the intervention, the post-test was administered under the same standardized conditions as the pre-test. The final step illustrates the data analysis workflow, including whole-sample analyses and analyses of the subsample with low initial concentration scores.

Eight 3rd-grade classes and four 2nd-grade classes from two primary schools in the southwest part of Germany participated in the recruitment process. The schools were selected because they were sufficiently large to provide multiple participating classes and also due to an established collaboration with the schools from previous research projects (convenience sample). The schools were located in an urban area and served a highly heterogeneous student population spanning both low and high socioeconomic backgrounds. Out of the 289 eligible children, 264 received approval to participate from their legal guardians. Children who had sick days during the pre- and post-tests and/or failed to meet 85% attendance rate for the intervention were excluded from the analysis. Approximately 40% of the children were excluded due to seasonal flu and COVID outbreaks during the testing period. Data from 157 children (81 boys, 76 girls) were finally analysed (see Fig. 1).

Approximately 70% of the participating children in each class had a migration background, and although German was the language of instruction and all children were able to fully understand the instructions and questions used in this project, many are bilingual and speak another language at home. None of them had prior experience with similar experiments. None of them had musculoskeletal and neurological disorders. Informed consent was obtained from both the parents or legal guardians and the children themselves.

The experimental procedures adhered to the ethical guidelines of the Declaration of Helsinki, as most recently revised in Fortaleza, as well as to the ethical standards established by the German Psychological Society (DGPs) and the American Psychological Association (APA). Explicit approval from the university’s ethical review board was not required for this study. In Germany, ethics committee approval is legally mandated only for specific types of research, such as studies involving medical products, therapeutic interventions, or research conducted by medical professionals in clinical contexts. School-based research, by contrast, is regulated through administrative procedures overseen by the local educational authorities. In the present study, all procedures were reviewed and approved by the responsible school authorities; therefore, an additional approval from the university ethics committee was not necessary.

### Groups

The study participants were divided into two three active intervention groups: balance training (BALANCE) involving moderate aerobic and anaerobic exercises (CARDIO), and cognitive training without physical activity (MENTAL). Children were class-wise and randomly assigned to these groups. BALANCE included 49 children (24 s graders, 25 third graders, 21 boys, 28 girls, average age 8.2 ± 0.68 years), CARDIO involved 49 children (16 s graders, 33 third graders, 27 boys, 22 girls, average age 8.3 ± 0.7 years), and MENTAL included 59 children (14 s graders, 45 third graders, 33 boys, 26 girls, average age 8.3 ± 0.62 years).

### Procedure

The study followed a cluster-randomized, three-arm, pre–post intervention design in which entire classes were randomly assigned to one of the three intervention groups (see Fig. 1 for overview). The study began with a pre-test conducted 5 days before the intervention started. On this day, assessments aimed at evaluating cognitive function and mathematics achievement were administered.

The intervention lasted for 5 weeks. Training sessions were conducted every school day, from Monday to Friday, and each session lasted approximately 15 min. In the majority of the cases (90%), these sessions were held at the beginning of mathematics lessons. However, in some school classes without daily mathematics lessons, the intervention took place also during other subjects, typically during general science or German lessons.

Post-tests assessing cognitive function and mathematics achievement were conducted 7 days after the final intervention session. Both pre- and post-tests were administered under identical conditions, in the same classrooms, at comparable late-morning times, and following standardized instructions, to ensure comparability across measurement occasions.

Testing procedures were carried out by the first author (CL), a co-author (BL), and six Master’s students who received standardized training prior to data collection. Two Master’s students were responsible for administering the KoKi concentration test at both measurement points, while two different students administered the mathematics achievement tests. All studied the respective test manuals and practiced the complete administration procedures together with the first author to ensure consistency. Additional Master’s students supported the testing sessions by distributing materials and monitoring task completion.

The classroom interventions were delivered by the same six Master’s students. Prior to the intervention phase, all received joint training on the content and timing of the three intervention formats (CARDIO, BALANCE, MENTAL), including detailed session plans and standardized instructions. During the intervention period, instructors followed a fixed rotation schedule across training days and both schools: on each day, two students visited all participating classes and implemented all three intervention types, with instructor pairs alternating across days. This procedure was intended to minimize systematic instructor-related effects.

Although formal fidelity checklists or quantitative adherence measures were not employed, instructors adhered to predefined session structures (e.g., station number, timing, exercise duration, and sequence) for each intervention type. Instructors communicated regularly with the first author throughout the intervention period to clarify procedures and address practical issues. No systematic deviations from the planned intervention protocols were reported. Minor situational adjustments (e.g., due to classroom space constraints) were made as needed, but the core elements and duration of the interventions were maintained across classes.

### Mathematics classes

To ensure that all participating classes covered comparable mathematical content during the intervention period, the teachers from the two participating schools coordinated their lesson planning. They discussed the specific exercises and teaching materials they intended to use, and the content to be taught was additionally predetermined by the official curriculum of the State of Baden-Württemberg (Bildungsplan).

### Interventions

#### Balance

The balance training program aimed at enhancing participants’ balance skills. Each session involved a rotation through seven to nine stations with different balance tasks. Each balance task was provided with easy-to-read instructions on how to perform the task. At the beginning of the training sessions, participants were initially grouped in subgroups of three to five children, and these subgroup assignments were consistently maintained throughout the session. An interval timer regulated the session pace, providing auditory cues for effective time management. During each training session, participants engaged in 45 s of activity followed by a 30- to 45-s pause for transitioning between stations. This structured routine persisted throughout the sessions, culminating in a total duration of 13 to 15 training minutes per session.

The overarching theme of the training program was to immerse children in a simulated circus environment, acquainting them with various acrobatic practices and skills. As participants progressed through the program, they encountered balance tasks of increasing difficulty. The tasks at each station were designed to challenge and improve balance control. Balance activities included tasks such as single-leg stance, walking on a line, and balancing on instable surfaces such as balance boards and pads. Children were encouraged to incrementally increase the difficulty level as they felt more confident in mastering the exercises, when managing to stand securely with minimal sway. Additionally, apart from self-monitoring, the instructors who supervised the children were adjusting the difficulty level on an individual basis. For example, if a child proficiently mastered balancing on a balance board with two legs, the child was asked to additionally balance a flat tray with both hands with a table tennis ball on top, preventing the ball from dropping. If this so-called dual task was mastered, the child further progressed by performing the same task standing only on one leg; first without the secondary task and later on with the dual-task.

#### Cardio

The CARDIO program was designed to improve participants’ aerobic and anaerobic fitness levels through a variety of exercises. Each training session included a circuit of seven to nine distinct stations with a focus on activating the cardiovascular system. Participants were organized into subgroups of three to five students at the start of each session and remained in these subgroups throughout the session. An interval timer regulated the session’s tempo, signalling transitions between stations. Each station involved 45 s of physical activity, followed by a short 30- to 45-s break before moving to the next station. This structure resulted in each session lasting between 13 and 15 min.

The overarching theme of the training program simulated an environment preparing children for the Olympics. The exercises covered a range of activities, including cardiovascular drills, agility drills, and plyometric movements like burpees, mountain climbers, and jumping jacks. Children were encouraged to perform each exercise with high intensity and received verbal encouragement from the experimenter during activity periods. This focus on intensity signalled progression during training, motivating children to consistently put forth their best effort.

#### Mental

The mental training program was developed with the objective of enhancing children’s selective attention abilities, complemented by integrated relaxation and mindfulness components. Cognitive exercises targeting selective attention included tasks such as painting from memory, auditory discrimination, numerical and lexical recognition, and narrative comprehension. The exercises were crafted to facilitate learning without the risk of reaching a performance ceiling. Concurrently, the program incorporated relaxation and mindfulness strategies through activities like meditation sessions and tactile stimulation from a partner.

These exercises were administered both individually and in collaborative settings, within the structured environment of a classroom under the guidance of the instructors. Each session adhered to a systematic schedule, allocating an average of 8 to 9 min for selective attention-focused exercises, succeeded by approximately 5 to 7 min dedicated to relaxation and mindfulness practices, maintaining a consistent sequential order throughout the program sessions. The exercises were systematically rotated during the comprehensive five-week training regimen, ensuring that no exercise was repeated within a timeframe of less than three days.

### Measurements in the pre- and post-test

#### Cognitive function

The KoKi test (Konzentrationstest für Kinder; test for concentration for children)^31^ was used as validated tool to objectively assess the concentration abilities of the children. The test is similar to the d2-R test^32^ but includes child-friendly drawings of dogs, horses, and wizards. The test comprises 8 pages with 100 images each, featuring rows of dogs and horses. Children are required to cross out dogs looking towards a sausage at the beginning or end of each row (target stimuli) while ignoring other distractors including “enchanted” horses with unusual features like wings, antlers or missing legs. These specific distractors are designed to capture attention and making it more challenging to ignore them. The KoKi test is highly reliable with internal consistency coefficients ranging from 0.83 to 0.96 (Cronbach’s Alpha). It also demonstrates construct validity by correlating with other established tests measuring similar constructs: the variable concentration of the KoKi significantly correlates with the variable concentration (0.69) of the d2-R test and the variable selective attention (0.58) of the Wechsler Intelligence and Development Scales^33^. Normative values for the test are based on a sample of 790 children in grades 1 to 4, with separate norms provided for different age groups. The test administration, including instructions and practice exercises, took approximately 15 min, while the test itself lasts 5 min and 20 s.

#### Mathematics achievement

We developed an assessment to measure the learning progress in a specific area of elementary school mathematics covered during the 5-week intervention. This test was administered before and after the intervention, thus it had to be ensured that children could at least partially solve it prior to the in-class introduction of the topic and that it would not be too easy afterward. To identify suitable topics for assessment we consulted the mathematics teachers from all participating classes. Based on their input, we selected subtraction of two-digit numbers (grade 2) and multiplication of two-digit numbers (grade 3).

Since existing standardized tests do not adequately cover these content areas, we developed a custom test for grades 2 and 3. The test consisted of two parts: a simple section included single-digit subtraction (grade 2) or multiplication (grade 3), and the difficult section included two-digit subtraction or multiplication. Strategies for two-digit calculations are based on knowledge about single-digit calculations and the decimal number system^30^, thus making the tasks in the difficult section accessible even before formal instruction. Hence, as children already had experience with subtraction and multiplication of single-digit numbers we could effectively assess their skills at the pre-test stage, too. While pupils may face challenges with these tasks prior to the intervention, we expected that as they engaged with two-digit calculations in class, they would not only improve their skills on two-digit calculations, but also become faster at one-digit calculations, because those are necessarily applied in two-digit problems.

The assessment was designed by an expert in mathematics education^34,35^ based on pedagogical content knowledge on task difficulty and calculation strategies, such as carrying over tens, handling zeros, working with whole tens, and performing carries. To ensure the test’s effectiveness and psychometric adequacy, we conducted pilot testing with children from two separate classes (eight 2nd graders and seven 3rd graders) prior to the main study. The pilot evaluation examined (a) whether children were able to meaningfully engage with both the easy and the difficult parts of the assessment, (b) whether ceiling or floor effects were present, and (c) whether item-level variation corresponded to the known performance differences among the participating children. The pilot testing confirmed that the test content was appropriate for both grade levels, that neither section exhibited saturation effects, and that the items produced sufficient variability to capture meaningful differences in children’s known mathematical performance. Although formal test–retest reliability was not conducted, the absence of ceiling or floor effects and the alignment between item difficulty and expected ability levels support the suitability of the assessment for detecting learning gains. These pilot procedures informed the finalization of the test and provided preliminary evidence of its validity and reliability for use in the main study.

#### Social validity

To assess the social validity of the study, mathematics teachers from the participating classes were asked to provide general feedback on the feasibility and usefulness of the interventions. The questioning of the teachers occurred 2 weeks after the post-test.

Social validity refers to the acceptability of the intervention by the recipients, in this case, teachers, and whether the intervention could make a meaningful difference in their opinion. Interventions that are considered socially significant (i.e., desirable and acceptable) are more likely to be adopted by teachers.

### Data analysis

#### Cognitive function

Concentration and accuracy were derived from the test results as normalized values (norming sample from the validation study of the KoKi, *N* = 790) aligned with the respective age cohort, and treated as dependent variables.

Concentration is defined as the number of processed test objects (regardless of whether they are solved correctly or incorrectly) minus the total number of errors, which encompasses both omission and confusion errors. This measure exhibits strong convergent construct validity with the concept of “selective attention” as outlined in the Intelligence and Development Scales^33^. Accuracy is calculated by dividing the concentration score by the number of processed test objects.

Normalized scores above 95 points and below 105 points are considered indicative of average test performance.

#### Mathematics achievement

The success rate for both the simple and difficult section of the test was calculated as the percentage of correctly solved maths problems relative to the total number of problems presented, and these rates were treated as dependent variables.

### Identifying students with lower concentration achievement

In the validation study of the KoKi test, children with ADHD exhibited concentration performance rates that were at least 0.58 standard deviations below the mean of their peers without ADHD^31^. We applied this criterion to our pre-test data, identifying children whose concentration scores were at least 0.58 standard deviations below the overall mean. This approach created a subsample to evaluate whether our interventions affected concentration differently in this specific group with reduced concentration.

### Statistical analyses

Whole Sample: Normality of the dependent variables was assessed using the Kolmogorov-Smirnov test; all variables were normally distributed. Dependent variables were therefore analysed using repeated-measures ANOVAs with the between-subject factor GROUP (BALANCE, CARDIO, MENTAL) and the within-subject factor TIME (pre-test, post-test). In cases where the assumption of sphericity was violated, Greenhouse-Geisser-corrected values are reported based on Mauchly’s test of sphericity. Effect sizes are reported as partial eta squared (η^2^) according to Bakeman^36^. In addition to p-values and effect sizes, estimated pre-post change scores with 95% confidence intervals are reported to quantify effect magnitude and precision.

Subsample: For dependent variables, change scores were calculated by subtracting pre-test values from post-test values for each participant, and group differences in these change scores were analysed using Kruskal-Wallis tests. Effect sizes are reported as epsilon squared (ε^2^), and 95% confidence intervals are provided for Hodges-Lehmann estimates of median group differences, estimating the precision of the observed effects. Because this subsample was defined post hoc, all corresponding analyses are considered exploratory.

Equal distribution of sex between all groups was tested with a Chi-squared test. The level of significance was set to *p* < .05 for all tests. Data analyses were performed using R programming language and R studio software (Posit, Boston). Figures were designed with R programming language and R studio software.

## Results

### Cognitive function

(i) Whole sample.

**Fig. 2:**
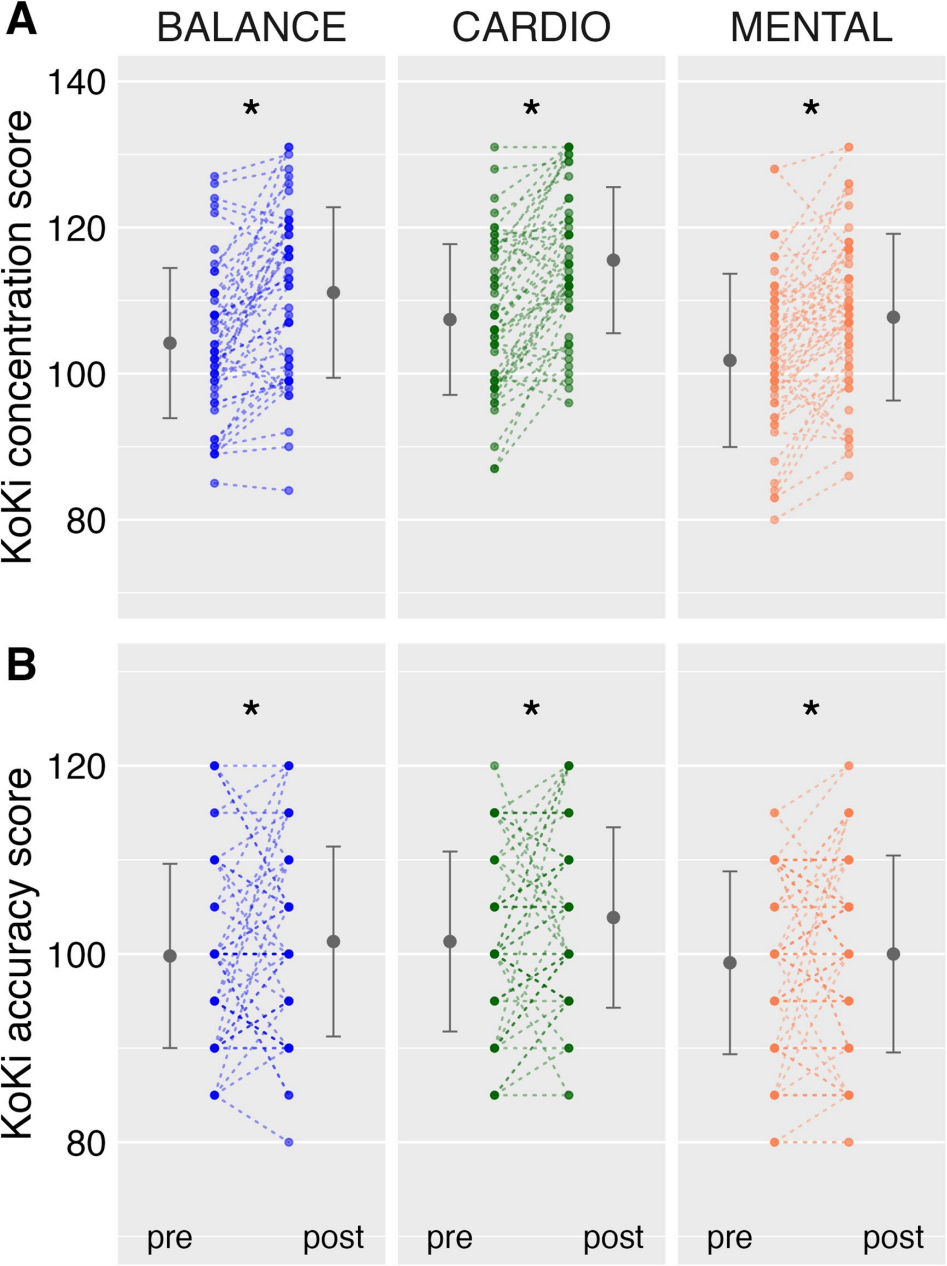
Displays the data on cognitive function derived from the KoKi test, specifically for concentration (**A**) and accuracy (**B**) with age-appropriate normalized test scores for both the pre- and post-test assessments. A score of between 95 and 105 serves as a reference point, indicating the average performance level of the comparison group utilized for test validation, referred to as age norms. Outer large dots signify group mean values, with vertical lines representing the standard deviation of the grand mean. Inner smaller dots depict individual data points, and lines are used to connect the pre- and post-values of each child. Notably, darker colors of the smaller dots denote instances where two or more children share the same values. Asterisk indicate significant results.

For the concentration measure of the KoKi test, the repeated-measures ANOVA revealed a significant main effect of TIME (F_1(154)_ = 164.39, *p* < .001, η^2^ = 0.12) and a significant main effect of GROUP (F_2(154)_ = 4.59, *p* < .01, η^2^ = 0.05), while the TIME × GROUP interaction was not significant. This pattern indicates that concentration values increased from pre- to post-test across all groups, with overall performance levels differing between groups. Pre-post changes showed increases in concentration for all three groups: BALANCE (Δ = 7.00, 95% CI [0.80, 13.20]), CARDIO (Δ = 8.44, 95% CI [2.42, 14.47]), and MENTAL (Δ = 6.07, 95% CI [0.39, 11.74]) (Fig. 2).

For accuracy, the ANOVA revealed a significant main effect of TIME (F_1(154)_ = 5.63, *p* < .05, η^2^ < 0.01), indicating a general increase in response accuracy from pre- to post-test. However, neither the main effect of GROUP (F_2(154)_ = 1.99, *p* = .14, η^2^ = 0.02) nor the TIME × GROUP interaction (F_2(154)_ = 0.15, *p* = .86, η^2^ < 0.01) reached significance. Pre-post changes in accuracy were small and accompanied by wide confidence intervals for all groups: BALANCE (Δ = 1.67, 95% CI [− 3.81, 7.14]), CARDIO (Δ = 2.69, 95% CI [− 2.64, 8.01]), and MENTAL (Δ = 0.82, 95% CI [− 4.19, 5.83]) (Fig. 2). The overlap of confidence intervals with zero indicates considerable variability among students.

(ii) Subsample with lower concentration values.

For the concentration measure, the Kruskal–Wallis test indicated small overall group differences in change scores, although the test did not reach conventional levels of statistical significance (χ^2^(2) = 5.57, *p* = .06; ε^2^ = 0.10). Pairwise comparisons based on Hodges-Lehmann estimates with 95% confidence intervals showed median improvements in the BALANCE group compared with the CARDIO group (HL = 7.0, 95% CI [1.0, 13.0]). In contrast, differences between BALANCE and MENTAL were smaller and uncertain, with confidence intervals including zero (HL = 3.0, 95% CI [− 3.0, 9.0]). Similarly, the comparison between CARDIO and MENTAL yielded confidence intervals spanning zero (HL = − 4.0, 95% CI [− 10.0, 2.0]). Overall, these findings suggest heterogeneous gains in concentration scores across intervention groups (Fig. 4).

For the accuracy measure, the Kruskal-Wallis test revealed no meaningful group differences in change scores (χ^2^(2) = 3.34, *p* = .18; ε^2^ = 0.06). Consistently, pairwise Hodges–Lehmann estimates indicated small median differences between groups, with all 95% confidence intervals including zero (e.g., BALANCE vs. CARDIO: HL = 2.0, 95% CI [− 2.0, 6.0]) (Fig. 4).

### Mathematics achievement.

(i) Whole sample.

**Fig. 3:**
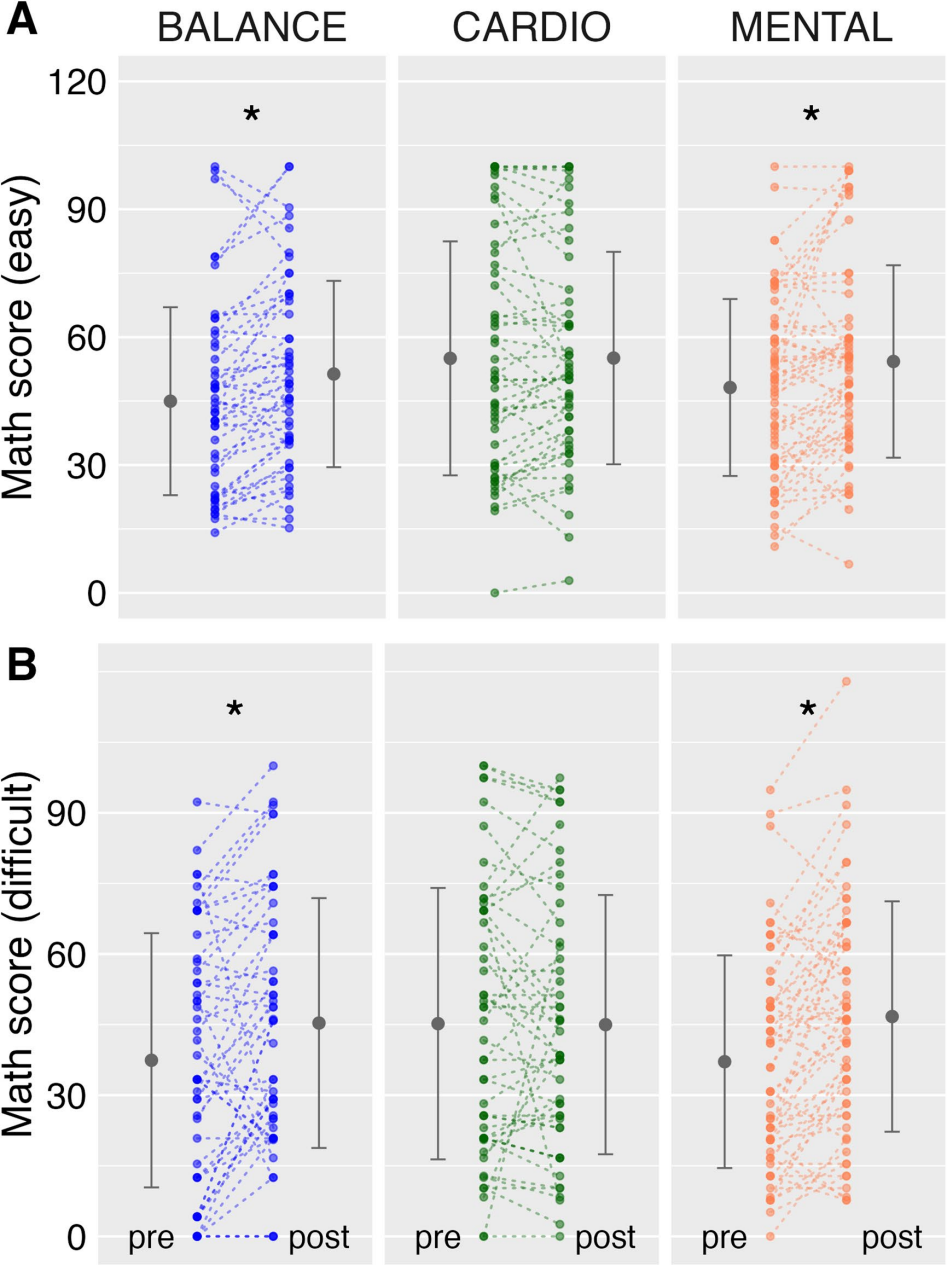
Illustrates the test results on mathematics achievement, namely the success rate (percentage of correctly solved tasks) for the easy section (**A**) and the difficult section (**B**) of the test, for both the pre- and post-test. Group mean values are depicted by outer large dots, with vertical lines indicating the standard deviation of the grand mean. Individual data points are represented by inner smaller dots, and lines connect the pre- and post-values of each child. It’s important to note that darker colors of the smaller dots signify instances where multiple children share the same values. Asterisk indicate significant results.

For the easy section of the mathematics test, the repeated-measures ANOVA revealed a significant main effect of TIME (F_1(154)_ = 19.67, *p* < .001, η^2^ = 0.01) and a significant GROUP × TIME interaction (F_2(154)_ = 4.98, *p* < .05, η^2^ < 0.01), while the main effect of GROUP was not significant (F_2(154)_ = 1.24, *p* = .29, η^2^ = 0.01). These results indicate that performance increased over time, but that the magnitude of change differed between groups. Pre-post changes showed mean improvements in the BALANCE group (Δ = 5.66, 95% CI [− 3.98, 15.31]) and the MENTAL group (Δ = 6.22, 95% CI [− 2.85, 15.28]), whereas the CARDIO group showed no change (Δ = 0.04, 95% CI [− 9.43, 9.50]) (Fig. 3). Confidence intervals were wide and overlapped zero in all groups, indicating variability in individual change scores.

For the difficult section of the test, the ANOVA likewise revealed a significant main effect of TIME (F_1(154)_ = 14.26, *p* < .001, η^2^ = 0.01) and a significant GROUP × TIME interaction (F_2(154)_ = 7.09, *p* < .001, η^2^ = 0.01), but no significant main effect of GROUP (F_2(154)_ = 0.35, *p* = .71, η^2^ < 0.01). This pattern indicates that changes over time differed across groups. Pre-post changes yielded mean improvements in the MENTAL group (Δ = 3.28, 95% CI [− 0.43, 7.00]) and the BALANCE group (Δ = 1.96, 95% CI [− 2.03, 5.95]), whereas the CARDIO group showed no improvement (Δ = −0.58, 95% CI [− 4.54, 3.37]) (Fig. 3).

(ii) Subsample with lower concentration values.

**Fig. 4:**
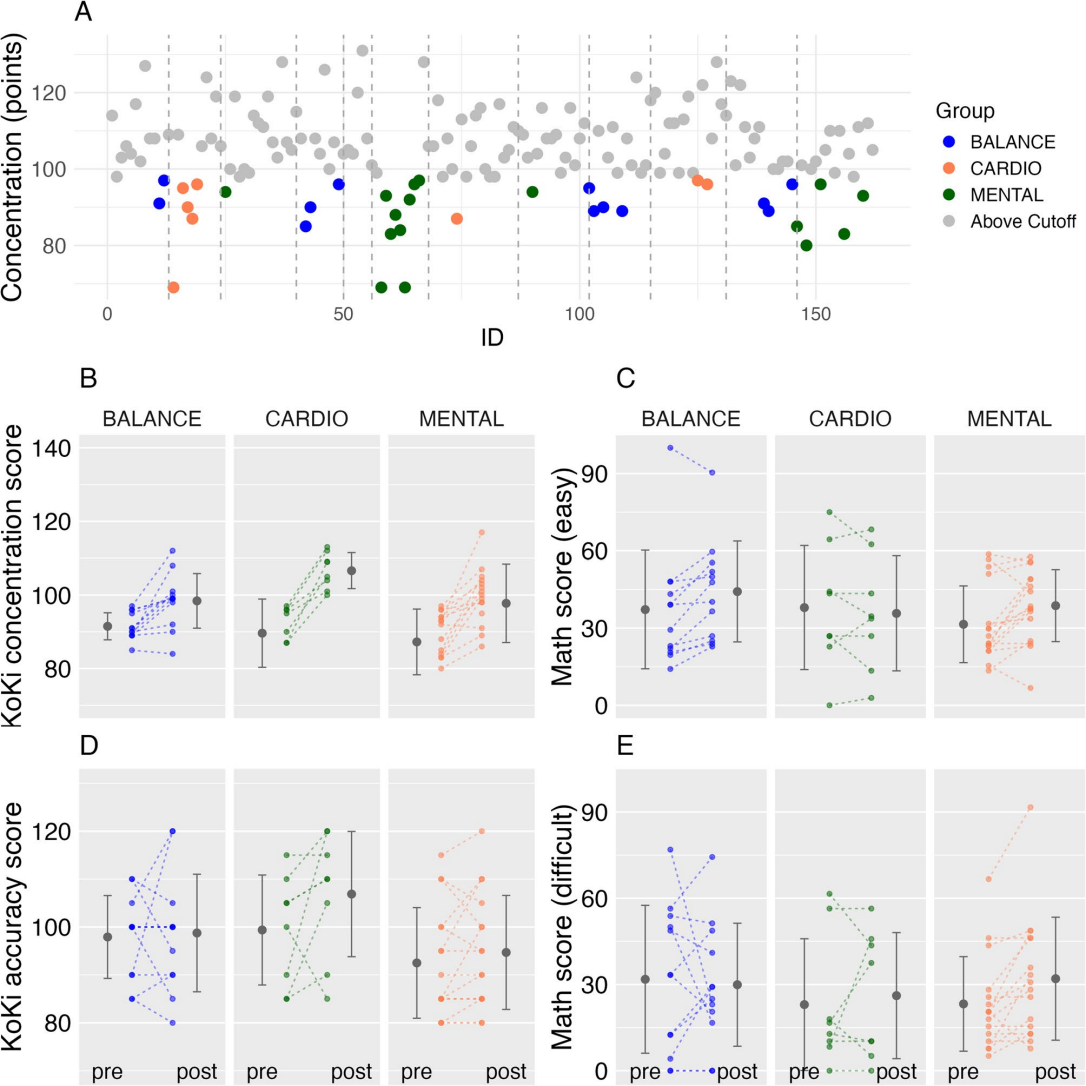
Presents the results for the subsample of children whose concentration levels are at least 0.58 standard deviations below the mean of the overall sample. Part (**A**) illustrates these children as colored points, categorized by their group affiliation, with dashed vertical lines indicating the different classes. Parts (**B**) and (**D**) depict the results from the KoKi test, specifically concentration (**B**) and accuracy (**D**). Parts (**C**) and (**E**) display the results for academic achievement for the easy (**C**) and difficult (**E**) sections of the test. Statistical analyses of the differences between pre- and post-test scores across groups are detailed in the Results section of the manuscript. Colored dots represent individual values, while gray dots and error bars indicate mean values and standard deviations.

For the easy section of the mathematics test, the Kruskal-Wallis test indicated small-to-moderate overall group differences in change scores (χ^2^(2) = 6.04, *p* = .04; ε^2^ = 0.11). Pairwise comparisons based on Hodges–Lehmann estimates with 95% confidence intervals showed that the BALANCE group exhibited larger median improvements than the CARDIO group (HL = 9.0, 95% CI [1.0, 17.0]). In contrast, the difference between BALANCE and MENTAL was negligible, with confidence intervals spanning zero (HL = 0.0, 95% CI [− 9.0, 7.0]). Comparisons between CARDIO and MENTAL suggested lower gains in the CARDIO group; however, the corresponding confidence interval included zero (HL = − 11.0, 95% CI [− 20.0, − 0.0]). Taken together, these results indicate heterogeneity in improvement across groups, with an increase observed for the BALANCE group relative to CARDIO, while differences involving the MENTAL group remain uncertain (Fig. 4).

For the difficult section of the mathematics test, the Kruskal-Wallis test revealed no meaningful group differences in change scores (χ^2^(2) = 2.50, *p* = .287; ε^2^ = 0.05). Consistently, pairwise Hodges–Lehmann estimates showed small median differences between all groups, with all 95% confidence intervals including zero (e.g., BALANCE vs. CARDIO: HL = 0.0, 95% CI [− 6.0, 6.0]) (Fig. 4).

### Group differences with respect to age and sex

There were no significant differences in age between the groups (F(2,154) = 0.66, *p* = .52, ηp^2^ < 0.01). For sex, the Chi-squared test was not significant (X2 (df = 2, *N* = 314) = 2.18, *p* = .34), indicating that sex and group are independent.

### Social validity

Teachers’ responses are summarized in Table 1. The responses varied significantly and addressed different aspects, concerned with tasks, organization, and individuals involved in conducting the experiments and interventions. Notably, three of the four teachers in the CARDIO group described the students as agitated, indicating that they observed the intervention had an unsettling effect on the students.

**Table 1:** Teachers’ responses concerning our question to the feasibility and usefulness of the intervention.

Grade	Group	English translation of teacher’s response
2	BALANCE	We were unable to observe any changes either during or after the project. The setup in the classroom was somewhat problematic, with little to very little space. The children mostly did not perform the exercises correctly.
2	BALANCE	The 2c mostly enjoyed the circus times and were quite motivated because the children truly believed they were training for a circus performance. I also noticed here who listens well in class and tries to perform exercises correctly, and who just does what they please.
3	BALANCE	The children enjoyed it. The movement session in the morning at school was beneficial for them. It was a pity that the children often rushed through exercises and paid little attention to performing them correctly on their own. Showing the exercises once at the beginning was not enough; I frequently went to the children and demonstrated the exercises again.
3	BALANCE	Diverse exercise formats, very motivating, difficult to implement alone in this format.
2	CARDIO	The class was more inattentive and agitated during this period. The training was not conducive to the learning atmosphere.
3	CARDIO	Motivating and age-appropriate tasks, station rotation requires a lot of structure/clarity. The intervention varied in structure depending on the instructor.
3	CARDIO	The class became very agitated. It was difficult to calm them down afterwards. Great selection of exercises. Prefer fewer options but with clear structure.
3	CARDIO	The children enjoyed participating and had fun. On some days, it was good for them to move around, but at times they were also very agitated after the training. I would have liked to do balance or concentration training.
2	MENTAL	Perhaps next there could be a study or program focusing on LISTENING skills?!
3	MENTAL	The children enjoyed the exercises and were motivated.
3	MENTAL	The class found it difficult to engage in the exercises. It takes a lot of experience to do such tasks with children at our school. BiSSZO [game] was great!
3	MENTAL	The project was very well implemented by the instructors. Great tasks/exercises.

## Discussion

### Summary of the results

This study examined the effects of different in-class interventions on cognitive function and mathematics achievement in primary school children. A systematic comparison of BALANCE, MENTAL, and CARDIO interventions showed that all three approaches were associated with increases in concentration levels. In contrast, improvements in mathematics achievement were observed in the BALANCE and MENTAL groups, whereas the CARDIO intervention was not associated with comparable gains.

In a subsample consisting of students with concentration levels below norm values, the BALANCE and MENTAL interventions were associated with small-to-moderate gains in mathematics achievement, whereas the CARDIO intervention was not. However, in contrast to the whole sample including all participants, significant improvements in mathematics achievement in this subsample with concentration deficits were observed only for the easier section of the test, not for the more challenging section.

### Effects on concentration levels

Our results show that concentration levels were higher in all three intervention groups. These findings confirm previous research on the benefits of CARDIO interventions^9,10^ and MENTAL interventions^27^. For the first time, however, this study displays similar effects for the BALANCE intervention. This was anticipated, as balance training has been shown to significantly enhance working memory and selective attention^23^ as well as memory and spatial cognition^29^.

### Effects on mathematics achievement

Only the BALANCE and MENTAL interventions were associated with significant improvements in mathematics achievement, evident in both, the easy and the difficult sections of the test. The easier section primarily consisted of exercises that children could solve using their prior knowledge, whereas the difficult section tested new knowledge acquired during the intervention period. A priori, we hypothesized that interventions would not only improve children’s fluency in solving the easier exercises but also enable them to tackle more of the challenging exercises. This expectation was supported by the observed results from the BALANCE and MENTAL groups, but not for the CARDIO group. Although BALANCE and MENTAL differ substantially in form—one being physically based, the other purely cognitive—they both target mechanisms that are known to support executive functioning. Balance training has previously been shown to enhance inhibitory control, selective attention and working memory^23,24,37^. Likewise, MENTAL exercises directly activate key executive functions such as working memory and selective attention, which are foundational for academic learning and mathematical problem-solving^27,38,39^. This may explain why BALANCE and MENTAL produced comparable gains in mathematics performance.

Previous studies have reported mixed outcomes regarding the impact of in-class aerobic and anaerobic exercise interventions, like those implemented with the CARDIO group, on mathematics performance^12,14,18–21^. In the present study, the absence of mathematics gains in the CARDIO group may be related to the high level of physiological arousal induced by the cardiovascular exercises. Three out of four teachers reported that students appeared overly stimulated and agitated following CARDIO sessions, which may have made it more difficult for them to settle down and concentrate during subsequent mathematics lessons. To our knowledge, this study is the first to demonstrate improved mathematics achievement following a BALANCE-based classroom intervention. A plausible explanation is that balance training enhanced students’ concentration without disrupting the classroom learning environment. While concentration levels also increased after the CARDIO intervention, balance exercises did not appear to induce excessive arousal or agitation. The combination of improved concentration and a calm post-intervention state may therefore be critical for supporting learning in mathematics lessons, where students must sustain attention while processing new content. This interpretation aligns with previous findings showing that balance training can enhance memory and spatial cognition in adults aged 19 to 65 years^29^.

In our subsample of students with lower concentration scores, we observed similar effects as in the main group: mathematics achievement improved in the BALANCE and MENTAL groups, but not in the CARDIO group. However, this improvement was only evident in the easier section of the mathematics test. Students in the subsample with lower concentration levels did not show gains across any of the three groups in the difficult math section, likely due to general challenges in mastering the material during the five-week period, which the interventions were unable to overcome.

### Considerations on the dose-response relationship

Building on previous findings, we argue that the dosage of the interventions in this study was sufficient to induce changes in cognitive function and academic achievement. Several studies have investigated the dose-response relationship between active breaks in the classroom and their impact on academic-related outcomes. Howie and colleagues^40^ examined different durations of active breaks (5, 10, and 20 min per break). They found that a 10-min active break significantly improved mathematics classroom behavior, and math scores were highest after the 10- and 20-min active break sessions. These positive effects on mathematics behaviour were later confirmed by Mavilidi and colleagues^41^. Janssen et al.^11^ focused on the intensity of active breaks, showing that moderate-intensity active breaks led to the best improvements in selective attention scores. Additionally, Altenburg et al. ^42^ investigated the frequency of active breaks, comparing once-per-day sessions to twice-per-day sessions. Their results indicated that children who participated in active breaks twice per day exhibited significantly better selective attention scores. These findings highlight the importance of considering the duration, intensity, and frequency of active breaks to optimize their effects on academic outcomes. According to these results, active breaks lasting 10 min or more at moderate intensity, performed at least once a day, had the largest positive impact on academic achievement.

### Limitations

Several limitations should be considered when interpreting the findings of this study.

First, the study relied on a convenience sample drawn from two primary schools with an existing collaboration, serving a highly heterogeneous student population. Although this facilitated implementation under authentic classroom conditions, it limits the generalizability of the findings to other school contexts, regions, or educational systems. Future studies may aim to replicate these findings in more diverse and representative samples.

Second, the study did not include blinding of participants, instructors, or assessors. Given the classroom-based nature of the interventions, blinding was not feasible. The absence of blinding may have introduced expectancy or performance effects, for example through differential motivation or engagement. Although standardized procedures and instructor rotation were used to minimize systematic bias, such influences cannot be fully ruled out.

Third, the study assessed outcomes only at two time points (pre-test and 7 days post-intervention). This short-term follow-up period precludes conclusions about the long-term sustainability of the observed effects on concentration and mathematics achievement. Longer follow-up intervals and repeated measurements would be necessary to determine whether the effects persist, diminish, or consolidate over time.

Fourth, the study lacked a true non-intervention (“business-as-usual”) control group. Because all groups received some form of active intervention, it is not possible to disentangle intervention-specific effects from general maturation, practice effects, or ongoing classroom instruction. While this design allowed for a direct comparison between different feasible classroom-based formats, future studies may include a passive control condition to more clearly isolate causal intervention effects.

Fifth, potential task-specific transfer effects may have influenced results in the MENTAL group. The cognitive exercises targeted selective attention and working memory, which overlap conceptually with the constructs assessed by the KoKi concentration test. Consequently, observed improvements may partly reflect near-transfer or practice-related effects rather than broader changes in cognitive functioning. Including additional outcome measures that assess distinct executive functions (e.g., inhibitory control or cognitive flexibility) would help address this issue.

Sixth, the study did not include objective measures of individual engagement or physical intensity during the BALANCE and CARDIO interventions. As a result, variation in children’s actual participation levels could not be quantified. Future research may incorporate objective or observational measures of participation and exertion.

Seventh, the study did not assess motivation, arousal, or affective states, which may moderate the relationship between active breaks and learning outcomes. In particular, teacher reports suggested heightened arousal following CARDIO sessions, which may have interfered with subsequent mathematics learning. Without direct measures, such interpretations remain speculative. Future studies should incorporate validated motivation scales, physiological indicators of arousal, and structured classroom observations. In addition, although sex was examined as a demographic variable, more nuanced measures of gender identity and motivational differences were beyond the scope of the present study and warrant future investigation.

### Practical implications

The practical implications of this study are grounded in the specific training parameters, daily 15-min sessions over five weeks in 2nd- and 3rd-grade mathematics classes, and highlight how schools might feasibly incorporate such interventions into everyday instructional routines.

First, the consistent improvements in concentration suggest that both physical (BALANCE, CARDIO) and non-physical (MENTAL) in-class activities can be integrated as short structured breaks to support students’ cognitive readiness for learning. From an educational perspective, considering the value of physical activity, balance-based tasks have particular potential for seamless integration into the daily school routine because they require minimal space, no specialized equipment, and can be implemented in short bursts without disrupting the classroom environment. Simple tasks such as stationary balance challenges (e.g., holding a pose while counting down from 15) can be embedded as “micro-breaks” before introducing cognitive content. These activities may foster attentional stability without elevating arousal to levels that interfere with subsequent classroom engagement, in contrast to the CARDIO intervention. Physical education teachers could support sustainable implementation in schools. They could train (non-physical education) teachers in safe and age-appropriate balance exercises, help design classroom-ready adaptations that require little space or supervision, and provide guidance on how to vary task difficulty to maintain student engagement.

The findings also highlight the potential usefulness of non-physical cognitive exercises, as demonstrated by the MENTAL intervention. Its effects on mathematics achievement were comparable to those of the BALANCE group. Thus, according to the outcomes in academic achievment, schools may select between physical and non-physical formats based on available resources, space, and teacher confidence.

Finally, the absence of gains in the CARDIO group raises an important practical consideration: the timing of more vigorous physical activities relative to core academic lessons. Teachers reported that students were overly excited and had difficulty settling afterward. Therefore, it may be more beneficial to schedule vigorous activity sessions after mathematics lessons, where they may support consolidation^43,44^ without impairing engagement during the learning phase^44^.

## Conclusion

In summary, the present study showed that the BALANCE and MENTAL interventions were associated with improvements in mathematics achievement among second- and third-grade primary school children, whereas the CARDIO intervention did not yield comparable academic benefits. Concentration levels were increased in all groups. Taken together, these findings suggest that both physical activity breaks and non-physical activity breaks can support students’ concentration levels, while benefits for mathematics achievement were observed for the BALANCE and MENTAL interventions. Given the predominantly sedentary nature of the school day and increasing health concerns related to physical inactivity in children^47–49^, physically active breaks such as balance training may provide additional benefits by combining cognitive gains with positive health-promoting effects.

## Data Availability

The datasets generated and analyzed during this study, with anonymized subject IDs, are available from the corresponding author upon reasonable request (contact via email).
